# Cleaning of Wastewater Using Crosslinked Poly(Acrylamide-*co*-Acrylic Acid) Hydrogels: Analysis of Rotatable Bonds, Binding Energy and Hydrogen Bonding

**DOI:** 10.3390/gels8030156

**Published:** 2022-03-03

**Authors:** Salah Hamri, Tewfik Bouchaour, Djahida Lerari, Zohra Bouberka, Philippe Supiot, Ulrich Maschke

**Affiliations:** 1 Center for Scientific and Technical Research in Physico-Chemical Analysis (CRAPC), BP 384, Industrial Zone, BouIsmaïl 42004, Algeria; salah_hamri@yahoo.fr (S.H.); lerari_zinai@yahoo.fr (D.L.); 2 Macromolecular Research Laboratory (LRM), Faculty of Sciences, Abou Bekr Belkaid University, BP 119, Tlemcen 13000, Algeria; bouchaour@yahoo.fr; 3 Laboratoire Physico-Chimie des Matériaux-Catalyse et Environnement (LPCMCE), Université des Sciences et de la Technologie d’Oran Mohamed Boudiaf (USTOMB), Oran 31000, Algeria; bouberkazohra@yahoo.fr; 4 CNRS, INRAE, Centrale Lille, UMR 8207—UMET—Unité Matériaux et Transformations, Université de Lille, 59000 Lille, France; philippe.supiot@univ-lille.fr

**Keywords:** wastewater, pollutant, dye, hydrogel, modeling, docking simulation

## Abstract

The discharge of untreated wastewater, often contaminated by harmful substances, such as industrially used dyes, can provoke environmental and health risks. Among various techniques, the adsorption of dyes, using three-dimensional (3D) networks consisting of hydrophilic polymers (hydrogels), represents a low-cost, clean, and efficient remediation method. Three industrially used dyes, Methylene Blue, Eosin, and Rose Bengal, were selected as models of pollutants. Poly(acrylamide) (poly(AM)) and poly(acrylamide-*co*-acrylic acid) (poly(AM-*co*-AA)) networks were chosen as adsorbent materials (hydrogels). These polymers were synthesized by crosslinking the photopolymerization of their respective monomer(s) in an aqueous medium under exposure to UV light. Experimental adsorption measurements revealed substantially higher dye uptakes for poly(AM-*co*-AA) compared to poly(AM) hydrogels. In this report, a theoretical model based on docking simulations was applied to analyze the conformation of polymers and pollutants in order to investigate some aspects of the adsorption process. In particular, hydrogen and halogen interactions were studied. The presence of strong hydrogen bonding plays a crucial role in the retention of dyes, whereas halogen bonding has a small or negligible effect on adsorption. An evaluation of binding energies allowed us to obtain information about the degree of affinity between polymers and dyes. The number of rotatable bonds in the copolymer exceeds those of poly(AM),meaning that poly(AM-*co*-AA) is revealed to be more suitable for obtaining a high retention rate for pollutants.

## 1. Introduction

Water is an important liquid for human beings, the universe, and all life existing on earth [1,2]. This liquid can be easily polluted by different dyes [3,4]. Both water and dyes are still largely used in the textile industry, meaning that the wastewater after production is a mixture of dyes and water. Unfortunately, the elimination of the wastewater often occurs through discharge into rivers and other effluents [5,6,7,8,9,10,11,12].

This negative situation has motivated many researchers to publish many reports in the field of the treatment of water polluted with dyes [13,14,15]. Several physical and chemical techniques have been developed to purify water from these compounds, including photocatalysis, oxidation, filtration, coagulation/flocculation, and adsorption [16,17,18,19,20]. In particular, adsorption processes have been studied intensively because of their low cost, easy access, and effective dye removal, in which the dissolved dye compounds adsorb on the surface of suitable adsorbents [21].

Many biodegradable materials and effective adsorbents obtained from natural resources have been used to remove dyes from aqueous solutions. Hydrogels were frequently applied for this purpose, consisting of a three-dimensional polymeric material with the capacity to uptake an important amount of water due to the presence of hydrophilic groups in their structure, such as –OH, –CONH, and –SO_3_H [22].

Copolymers based on acrylamide (AM) and acrylic acid (AA) have been applied to remove dyes. AM monomer is soluble in water, and linear poly(AM) finds many uses as water-soluble thickeners and flocculation agents [23,24,25], whereas AA represents the simplest unsaturated carboxylic acid. This colorless liquid is miscible with water, alcohols, ethers, and chloroform [26,27,28]. Solpan et al. [29] used (poly(AM-*co*-AA) hydrogels for the uptake of the cationic dyes, safranin-O and magenta. The diffusion of water and cationic dyes within hydrogels showed non-Fickian behavior. Corona-Rivera et al. [30] applied poly(AM-*co*-AA) crosslinked with N,N′-methylene bisacrylamide (NMBAM) for the removal of Remazol red dye from aqueous solutions, finding the maximum dye adsorption capacity for peculiar experimental conditions, with an adsorption mechanism well represented by the Langmuir model.

The diffusion of colored water inside the hydrogel depends on many factors, such as the dye structure and the functional groups on the polymer chains. The dye can generate an attraction through electrostatic interaction, which also represents an important key to removing dye from an aqueous medium, whereby a dye molecule and a receptor behave similarly to a ship and a harbor. In the field of biochemistry, the theory of docking was largely applied to study the interaction between ligand and protein, allowing us to explain the affinity between these components [31,32,33,34,35,36,37]. In this study, the interactions between the dyes and polymer networks were investigated. The docking method was applied to analyze different interactions, with the receptor and ligand representing the polymer matrix and dye, respectively. This simulation method has the advantage of enabling us to predict the preferred orientation of one molecule to a second one when bound to each other to form a stable complex [38,39,40]. Interestingly, this helps us to economize cost and time of research work.

In a previous paper [41], the interaction between a polymer based on HEMA monomer and Eosin Y (EY) as a pollutant was discussed. It was found that the theoretical prediction correlates well with experimental results. In the literature, some authors report on poly(AM-*co*-AA) crosslinked with NMBAM [42,43,44,45,46]. In this work, AM and AA were copolymerized and crosslinked with HDDA since it contributes to the high level of conversion of acrylic double bonds [47,48]. Under the UV-visible light exposure in the presence of a suitable photoinitiator (Darocur 1173), a chemically crosslinked three-dimensional copolymer was successfully obtained. In contrast to thermal polymerization, which often requires elevated temperatures, photopolymerization can be performed at room temperature [49]. In most reports on poly(AA), thermal polymerization was applied using a source of free radicals, together with a chemical stabilizer, such as ammonium persulfate and tetramethylethylenediamine [50,51,52]. This method has disadvantages such as long polymerization times, unstable and toxic reagents, and tedious preparation steps. On the other hand, photopolymerization using an initiator sensitive to light represents a quicker method that is less tedious and less toxic. The field of the exploitation of these polymeric materials is, thus, enlarged to applications in which the elevation of temperature is not advised. The final properties of UV-polymerized gels depend on the UV-visible spectrum of the source, light intensity and uniformity, and exposure times [53].

The model dyes studied in this report were Rose Bengal (RB), EY, and Methylene Blue (MB), presenting anionic and cationic natures. These dyes are widely used in many applications, thus increasing the probability that they contribute to water pollution, since even small quantities can easily affect the water quality [54,55,56].

To understand the different interatomic interactions between dyes and polymers, the docking simulation method was exploited. Two model systems, crosslinked poly(AM)/dye and poly(AM-*co*-AA)/dye, were considered using Avogadro software. These model systems were all energy-minimized, and the conformation of polymer/dye systems was simulated using Auto-Dock Vina software [57,58], and then visualized and analyzed using UCSF Chimera.

## 2. Results and Discussion

### 2.1. Effect of Crosslinker Content on Equilibrium Swelling

To find out the optimal dye concentration for the retention study, the UV-visible spectra of the dyes were screened in the concentration range from 32 × 10^−3^ mg·mL^−1^ to 64 × 10^−3^ mg·mL^−1^. According to the obtained results (Figure S1), the spectra corresponding to 64 × 10^−3^ mg·mL^−1^ reveal saturation effects for all absorbance bands except those from RB. Electronic spectra associated with the lower concentration of 32 × 10^−3^ mg·mL^−1^ were acceptable; therefore, this concentration was chosen for the retention study.

**Figure 1:**
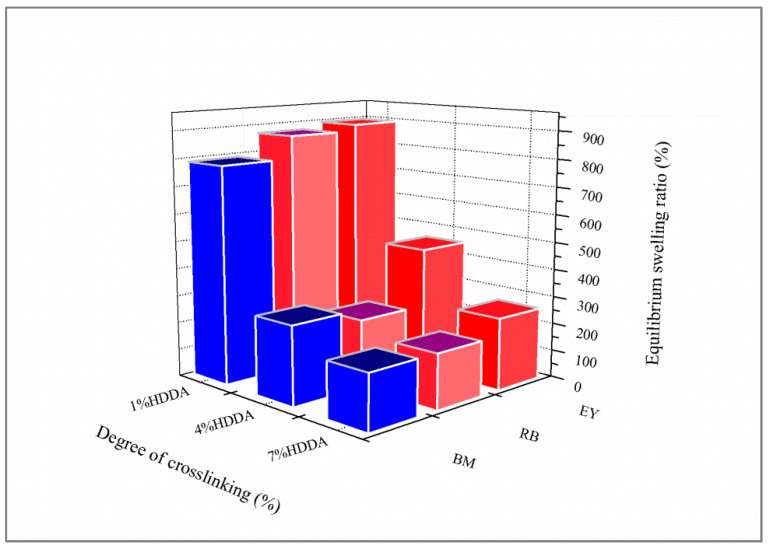
Effect of the composition of HDDA (wt%) on equilibrium swelling of poly(AM) hydrogel in dye solutions.

Figure 1 presents the evolution of poly(AM) swelling equilibrium versus the composition of a crosslinking agent (HDDA) for each dye solution. Equilibrium swelling data were remarkably increased by decreasing the crosslinker content. The best results of maximum equilibrium swelling were obtained with 1 wt% of HDDA. In this case, swelling values in solutions of RB, BM, and EY were found at around 870%, 850%, and 900%, respectively. The crosslinking density essentially governs the diffusion of the dyes in the polymer networks, as well as the swelling behavior.

**Figure 2:**
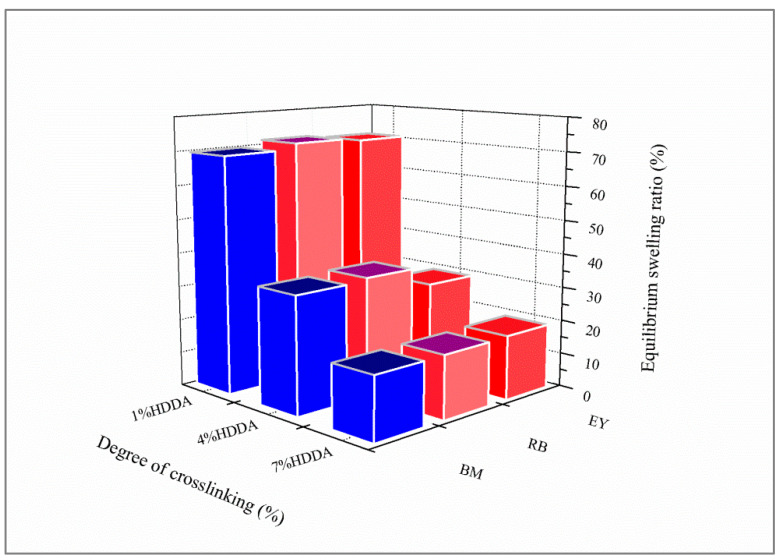
Effect of the composition of HDDA (wt%) on equilibrium swelling of poly(AM-*co*-AA) hydrogel in dye solutions.

Figure 2 presents the evolution of the swelling equilibria of poly(AM-*co*-AA) versus the composition of HDDA for each dye solution. The copolymer prepared with 1 wt% HDDA shows the highest equilibrium swelling with 70%, 72%, and 71% in solutions of BM, RB, and EY, respectively. In comparison with poly(AM), poly(AM-*co*-AA) presents a much lower equilibrium swelling due to the addition of AA units, thus increasing the crosslinking density. Swelling equilibrium values of 34%, 35%, and 29% were obtained for 4 wt% HDDA in solutions of BM, RB, and EY, respectively. For 7 wt% HDDA, the corresponding swelling data were 18%, 19%, and 19%.

### 2.2. Effect of Crosslinker Content on Absorbance

#### 2.2.1. Rose Bengal Dye

**Figure 3:**
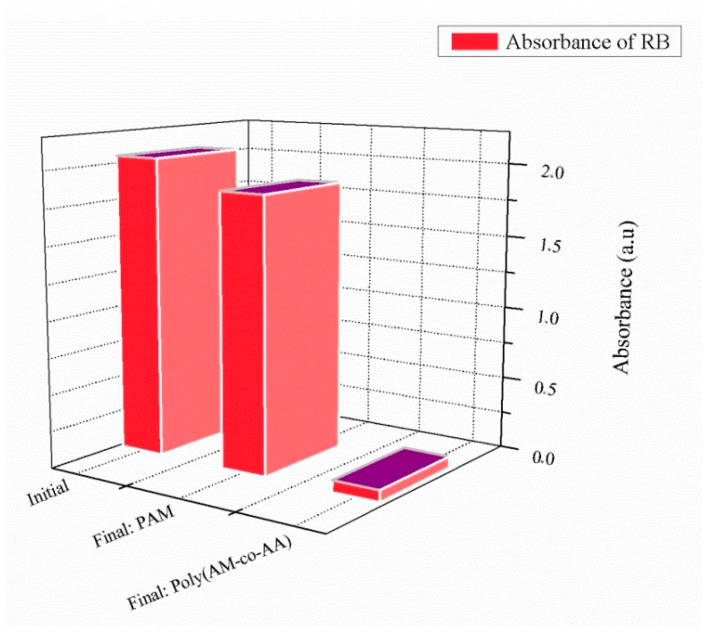
Retention behavior of RB in presence of crosslinked poly(AM) and poly(AM-*co*-AA) (1 wt% HDDA, after 24 h contact time).

Figure 3 illustrates the retention behavior of RB using poly(AM) and poly(AM-*co*-AA). A retention rate of about 7% for RB by poly(AM) was obtained, whereas 97% of RB was removed in the case of poly (AM-*co*-AA) (Figure S2).

The hydrogen bonding between chains of the neutral poly(AM) provokes physical crosslinking effects; the poly(AM) network is brittle and its glass transition was reported to be around 450 K [59,60].

From the Morse curve [61], considering large distances, the energy is zero (no interaction). This means that two atoms placed infinitely far away do not interact with each other, or they are not bonded to each other. At inter-nuclear distances in the order of the atomic diameter, attractive forces dominate. At smaller distances between two atoms, the force is repulsive and the energy of the two atoms is high.

The distance between atoms has, thus, an important effect on the interactions of the system. It was found that interactions can be classified as strong and medium for a distance interval of [2.5, 3.1] Å and [3.1, 3.55] Å, respectively, whereas distances between selected atoms greater than 3.55Å correspond to weak or non-existing interactions [41].

**Figure 4:**
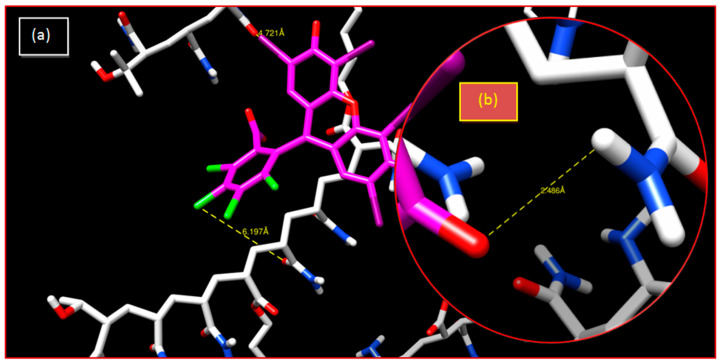
Crosslinked poly(AM)/RB system: (**a**) 3-D representation of results of the interaction; (**b**) enlargement of the hydrogen bonding interaction.

In the case of crosslinked poly(AM) hydrogel, the distances between chlorine and oxygen atoms are greater than 3.55 Å, which shows that the interactions are weak. The hydrogen bond with 2.48 Å represents a strong interaction, but the neutrality of the hydrogel cannot allow this bond to be constructed (Table 1 and Figure 4).

**Figure 5:**
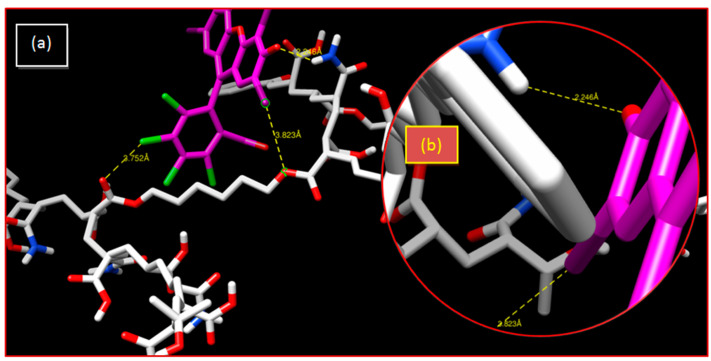
Crosslinked poly(AM-co-AA)/RB system: (**a**) 3-D representation of results of the interaction; (**b**) enlargement of the hydrogen bonding interaction.

In the crosslinked poly(AM-*co*-AA)/RB system, there are weak and average electrostatic interactions between the AA fraction and RB. The interaction between chlorine and iodine with oxygen is of a halogen type. The corresponding interatomic distance is higher than 3.55 Å, thus resulting in a weak interaction. Furthermore, the hydrogen bond with 2.24 Å represents a strong interaction, because the copolymer is charged in the aqueous medium and becomes a polyelectrolyte. Repulsion occurs between the negative charges of the AA parts and, consequently, the dye was retained by the strong hydrogen bond (O...H). The AA fraction of poly(AM-*co*-AA) has increased the retention percentage from 7% to 97%; therefore, it can be concluded that this copolymer effectively retains RB in an aqueous medium (Table 1 and Figure 5).

#### 2.2.2. Methylene Blue Dye

**Figure 6:**
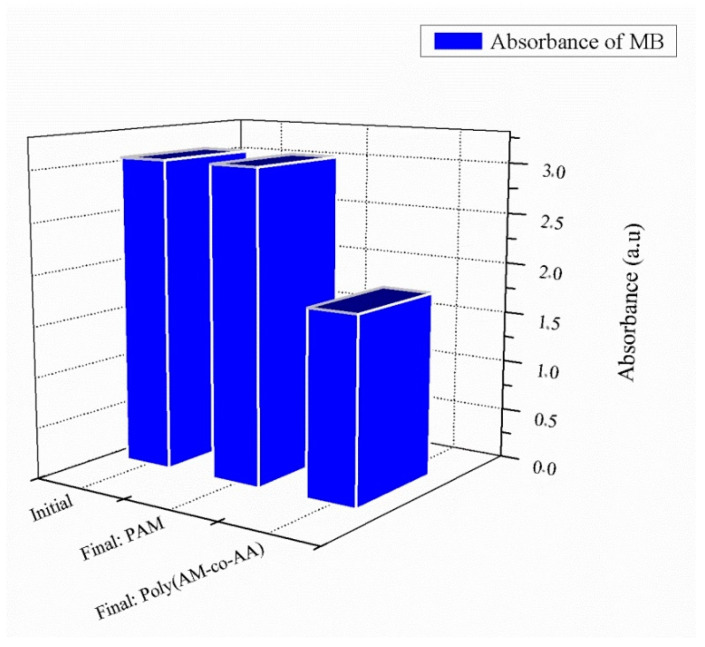
Retention of MB in presence of crosslinked poly(AM) and poly(AM-co-AA) hydrogels (1% wt HDDA, after 24 h contact time).

Figure 6 shows that crosslinked poly(AM) presents a negligible retention of MB (about 1%), whereas poly(AM-*co*-AA) removes MB at rates of 45% for a contact time of 24 h.

**Figure 7:**
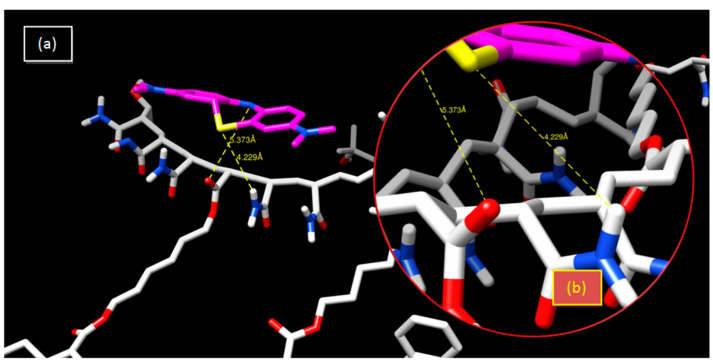
Crosslinked poly(AM)/MB system: (**a**)3-D representation of results of the interaction; (**b**) enlargement of the hydrogen bonding interaction.

In the neutral hydrogel, the interatomic distance between nitrogen and oxygen is 5.37 Å, which means that there is a weak attraction between these two atoms. A similar situation occurs between sulfur and hydrogen atoms (Table 2 and Figure 7). Initial and final spectra of the dye are shown in Figure S3.

**Figure 8:**
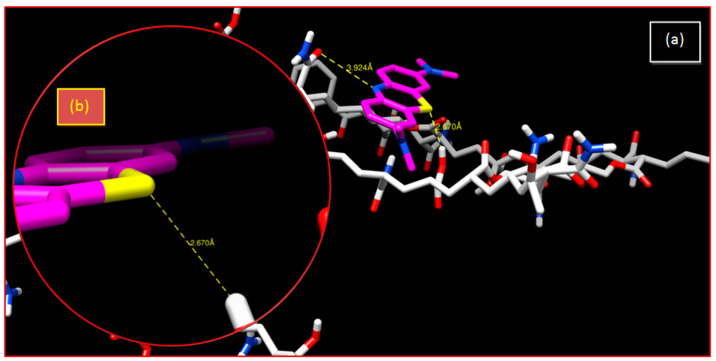
Crosslinked poly(AM-*co*-AA)/MB system: (**a**) 3-D representation of results of the interaction; (**b**) enlargement of the hydrogen bonding interaction.

Sulfur atoms have been known to participate in hydrogen bonds. It has been shown that the sulfur atom is a poor H-bond acceptor, but a moderately good H-bond donor [62]. In the copolymeric hydrogel, there is a hydrogen bond with an interatomic distance of 2.67 Å, which is considered to be a strong bond, facilitating an increase in the retention percentage from 1% to 45% (Table 2 and Figure 8).

#### 2.2.3. Eosin Y Dye

**Figure 9:**
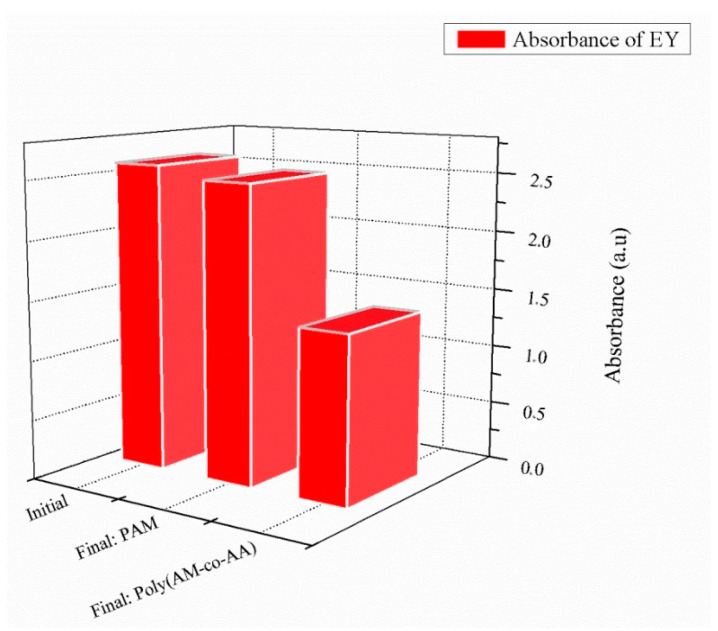
Retention of EY in the presence of crosslinked poly(AM) and poly(AM-*co*-AA) hydrogels (1% wt HDDA, after 24 h contact time).

Figure 9 reveals a very small adsorption effect of EY by poly(AM) (0.4%). Halogens participating in the halogen bonding of the investigated dyes include iodine (I) (present in RB), bromine (Br) (present in EY), and chlorine (Cl) (present in RB). These halogens are able to act as donors and follow the general trend of Cl < Br < I, with iodine normally forming the strongest interactions [63,64].

**Figure 10:**
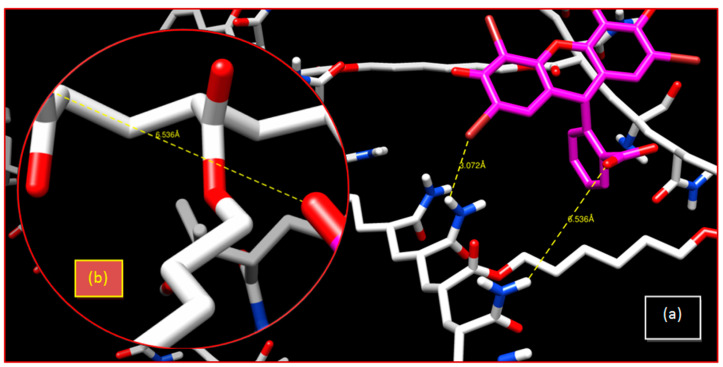
Crosslinked poly(AM)/EY system: (**a**) 3-D representation of results of the interaction; (**b**) enlargement of the hydrogen bonding interaction.

**Figure 11:**
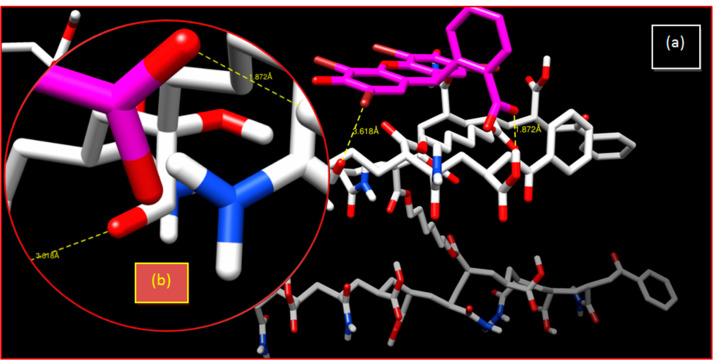
Crosslinked poly(AM-co-AA)/EY system: (**a**) 3-D representation of results of the interaction; (**b**) enlargement of the hydrogen bonding interaction.

For this neutral system (poly(AM)), there is a Br...O bond with an interatomic distance higher than 3.07 Å and a hydrogen bond with 6.53 Å (Table 3 and Figure 10) that qualify these bonds as weak bonds. Initial and final spectra are presented in Figure S4. A strong hydrogen bonding of poly(AM-*co*-AA) exists with 1.872 Å, which improves the retention of the MB dye (Figure 11).

**Figure 12:**
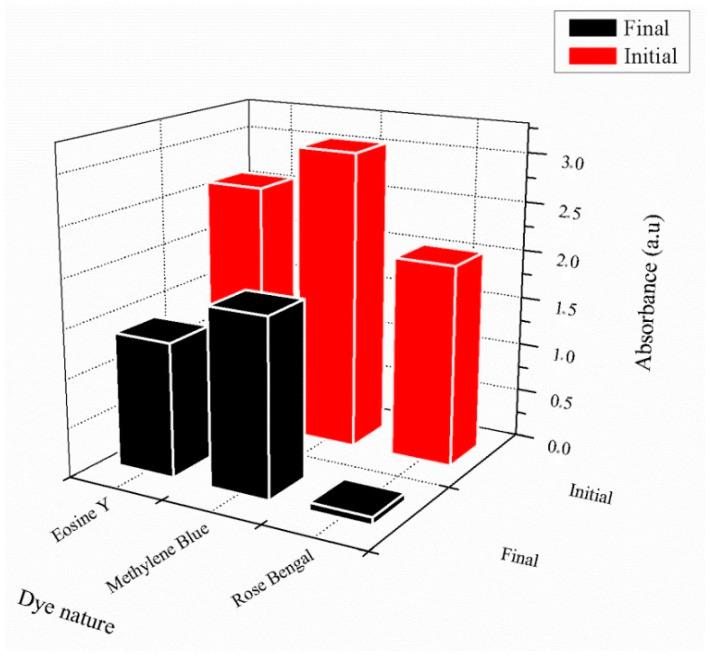
Illustration of the uptake efficiency for all dyes in presence of poly(AM-*co*-AA) hydrogel.

Figure 12 represents a summary of the adsorption results for poly(AM-*co*-AA) hydrogel, showing retention percentages of 45%, 50%, and 97% for MB, EY, and RB, respectively. Poly(AM-*co*-AA) functions, thus, with considerable efficiency, removing a high percentage of RB, though it is less effective for EY and MB. This difference can be explained by the different molecular structures and architectures of these dyes. Moreover, RB possesses more functional groups compared to EY and MB.

### 2.3. Binding Energy and Number of Rotatable Bonds Analysis

AutodockVina software allows us to determine the binding energies, which were used to evaluate if dyes could have stable complex interactions with polymeric hydrogels. The negative sign of the binding energy means that the dye was bound spontaneously without consuming energy. If the sign is positive, the binding occurs only if the required energy is available. Lower values of binding affinity correspond to a higher stability of polymer/dye complexes. Consequently, hydrogel/dye systems with the highest and lowest stability in Table 4 were poly(AM-*co*-AA)/RB and poly(AM)/MB, respectively.

**Figure 13:**
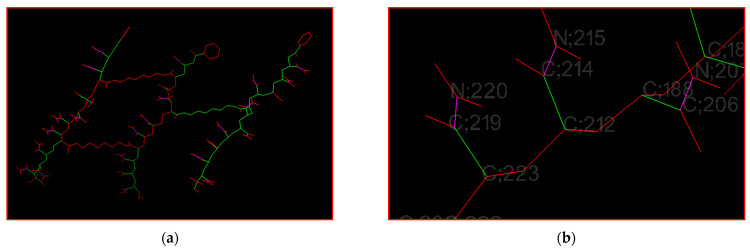
Rotatable bonds of the crosslinked poly(AM) model using Autodock tools: (**a**) 92 rotatable bonds, (**b**) one rotatable bond of the AM repetition unit. Green: rotatable, magenta: nonrotatable, red: unrotatable bond.

A rotatable bond is defined as any single non-ring bond attached to a non-terminal, non-hydrogen atom. In Figure 13a, presenting the crosslinked poly(AM) model using Autodock tools, most bonds were nonrotatable. When two HDDA units were very close, the crosslinker creates rigidity in the polymer network. The presence of a single crosslinking unit leads to more rotatable bonds. In Figure 13b, representing the AM repetition unit, we can see that C214–N215 bonds are nonrotatable; the same situation applies for C219–N220: amide C-N bonds present a high energy barrier for rotation [65,66]. C212–C214 and C223–C219 bonds are rotatable, i.e., the AM repetition unit possesses one rotatable bond.

**Figure 14:**
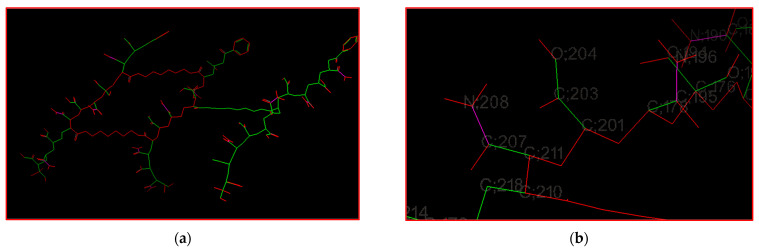
Rotatable bonds of the crosslinked poly(AM-*co*-AA) model using Autodock tools: (**a**) 104 rotatable bonds, (**b**) two rotatable bonds of the AA repetition unit. Green: rotatable, magenta: nonrotatable, red: unrotatable bond.

Figure 14a presents 104 rotatable bonds of the crosslinked poly(AM-*co*-AA) model, using Autodock tools. Figure 14b shows the AA repetition unit of poly(AM-*co*-AA), exhibiting two rotatable bonds, C201–C203 and C203–C204.

The crosslinked poly(AM) model presents 92 rotatable bonds whereas the crosslinked poly(AM-*co*-AA) presents 104 rotatable bonds (Table 5). This difference creates more conformations for the copolymer compared to the homopolymer, so that polymer–dye interactions are favored for crosslinked poly(AM-*co*-AA).

## 3. Conclusions

The UV photopolymerization technique in an aqueous medium was chosen to elaborate chemically crosslinked poly(AM) and poly(AM-*co*-AA) as dye adsorbent hydrogels. Experimental parameters, such as the optimal percentage of HDDA as a crosslinking agent, as well as the suitable dye concentration for analysis, were found to be1 wt% and 32 × 10^−3^ mg·mL^−1^, respectively. All dyes show negligible retention effects using the neutral poly(AM), and significant adsorption for the polyelectrolyte poly(AM-*co*-AA). It was found that 97% of RB was removed efficiently by the copolymer (MB: 45%, EY: 50%), which can be related to the presence of one more functional group compared to the other dyes, and also due to strong hydrogen bonding (O...H) with an interatomic distance of 2.24 Å, which plays a key role in interaction. As a consequence, this copolymer could be considered to be an efficient hydrogel with which to remove the considered dyes from a water medium.

The conformation of polymers and pollutants were analyzed via a docking simulation. Interestingly, halogen bonding could be neglected, whereas hydrogen bonding plays a key role for dye retention. The system composed of poly(AM-*co*-AA)/RB has a binding energy of −7.7 kcal/mol, which means that this system has the highest stability compared to the other investigated polymer/dye systems. An analysis of rotatable bonds shows that the AA repetition unit presents two rotatable bonds, whereas that of AM has one; therefore, poly(AM-*co*-AA) has more conformations than poly(AM), thus increasing dye retention.

## 4. Materials and Methods

### 4.1. Materials

The monomers used in this study were AM and AA (both from Sigma-Aldrich (Saint-Quentin-Fallavier, France), purity: 99%), the crosslinking agent was HDDA (from Cray Valley, Courbevoie, France), purity: 98%), and the photoinitiator was 2-hydroxy-2-methyl-1-phenyl-propane-1 (commercial designation: Darocur 1173) (from Ciba-Geigy, purity: 97%). RB (purity: 95%), EY (purity: 99%), and MB (purity: 70%) (all from Sigma-Aldrich) were applied as dyes. All products were used as received without purification. The chemical structures of the reagents are illustrated in Table 6. Abbreviations are given in Table S1.

### 4.2. Hydrogel Synthesis

First, the AM monomer was dissolved in distilled water. Then, 0.5 wt% of Darocur 1173 was added. To underline the crosslinker effect on the water uptake capacity of the obtained hydrogels, a set of three solutions with different percentages of HDDA (1 wt%, 4 wt%, and 7 wt%) was prepared. After steering for 24 h, the solutions were exposed to UV irradiation for 30 min, using a TL08 UV lamp, with a characteristic wavelength of λ = 365 nm and an intensity of 1.5 mW/cm^2^. For the sake of comparison, a hydrogel copolymer was generated. A stock solution of 50 wt%/50 wt% AM/AA was firstly prepared. In the second step, three solutions were prepared with different percentages of HDDA: 1 wt%, 4 wt%, and 7 wt%, with 98.5 wt%, 95.5 wt%, and 92.5 wt% of the AM/AA solution, respectively. Finally, Darocur 1173 as a photoinitiator was added to each of these solutions (0.5 wt%). All solutions were prepared at room temperature.

After the polymerization/crosslinking process (see also Figure S5), all samples were obtained in pellet form (sample thickness: 3 mm, diameter: 2.5 cm) and washed in distilled water to remove all remaining residue.

### 4.3. Dye Retention Experiments

The selected cylindrical hydrogel (1.5 g), as an adsorbent, was immersed in 32 × 10^−3^ mg·mL^−1^ of dye solution at room temperature (T = 23 °C) for 24 h. Then, the hydrogel was separated by filtration, and the residual concentration of the considered dye solution was introduced in a glass flask and evaluated using a dual-beam ultraviolet–visible spectrometer (Specord 200 plus, Analytik Jena, Jena, Germany).

### 4.4. Equilibrium Swelling Measurements

In order to underline the equilibrium swelling of the elaborated hydrogels, the sample was weighed in the dry state and then immersed in a dye solution for 24 h under stirring. Then, the sample was wiped with a filter paper to remove free liquid on the surface before being weighed. The degree of swelling was calculated according to Equation (1).

(1) $$\tau {\left(\%\right)}_{}=100\left(\frac{m_{t}-m_{0}}{m_{0}}\right)$$

where *τ*(%) represents the degree of swelling, *m_t_* stands for the weight of the swollen network at time *t*, and *m*_0_ is the weight of the initially dried network.

### 4.5. Model Proposition

Two model systems were proposed. The first one represents the crosslinked poly(AM)/HDDA system, based on three chains of poly(AM) containing ten units each. Chains were connected by three HDDA crosslinking nodes. The second model system, the crosslinked poly(AM-*co*-AA), was created similar to the first model. RB, EY, and MB were all presented in 3-D. All models were energy-minimized using auto-optimization with force field UFF and the steepest descent algorithm of the Avogadro software. The output simulation implies eight conformations; the best conformation of each hydrogel/dye system was illustrated based on their energy.

### 4.6. Software

**Figure 15:**
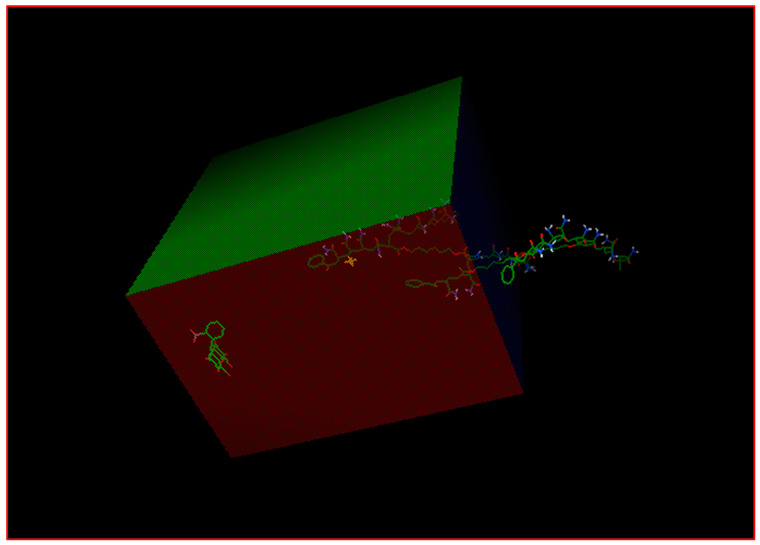
Grid box containing dye and polymer network using Autodock tools.

Avogadro version 4.8.6 was used to visualize and optimize the model systems. The files were saved in the molecule file format pdb. AutoDock version 1.5.6, a molecular modeling simulation software, represents a suite of automated docking tools. It is designed to predict how dyes bind to polymer networks; the grid box allows the user to limit the space of interaction analysis (Figure 15).

The simulation was conducted in dimensions of grid box points (x = y = z = 126 Å), and the grid box center dimensions were set as mentioned in Table S2. The other parameters were maintained and were used as defaults. Finally, the output file (log.txt) was analyzed, and the best docking results regarding binding energies were selected and investigated with other software programs. The Chimera calculation software UCSF version 1.5.3 was used to analyze interatomic distances.

## Figures and Tables

**Table 1:** Interatomic distances obtained from interactions of the two polymers with RB using the docking simulation method.

System	Bonds	Distance (Å)
Poly(AM)/RB	I...O	4.721
	C...O	6.197
	O...H	2.486
Poly(AM-*co*-AA)/RB	I...O	3.823
	Cl...O	3.752
	O...H	2.246

**Table 2:** Interatomic distances obtained from interactions of the two polymers with MB using the docking simulation method.

System	Bonds	Distance (Å)
Poly(AM)/MB	S...H	4.229
	N...O	5.373
Poly(AM-*co*-AA)/MB	S...H	2.670
	N...O	3.924

**Table 3:** Interatomic distances obtained from interactions of the two polymers with EY using the docking simulation method.

System	Bonds	Distance (Å)
Poly(AM)/EY	O...H	6.536
	Br...O	3.072
Poly(AM-*co*-AA)/EY	O...H	1.872
	Br...O	3.618

**Table 4:** Binding energies of polymer/dye systems.

Polymer/Dye	Binding Energy (kcal/mol)
Poly(AM)/RB	−7.0
Poly(AM)/EY	−5.2
Poly(AM)/MB	−4.1
Poly(AM-*co*-AA)/RB	−7.7
Poly(AM-*co*-AA)/EY	−5.1
Poly(AM-*co*-AA)/MB	−4.4

**Table 5:** Number of rotatable bonds of poly(AM)/HDDA, poly(AM-co-AA)/HDDA and dyes, obtained by Autodock software.

Product	Number of Rotatable Bonds
poly(AM/HDDA)	92
poly(AM-*co*-AA)/HDDA	104
AM repeat unit	01
AA repeat unit	02
RB	2
EY	2
MB	2

**Table 6:** Chemical structure of monomers, crosslinker, and dyes.

Name	Chemical Structure
Acrylamide (AM)	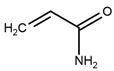
Acrylic acid (AA)	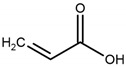
1,6-Hexanedioldiacrylate (HDDA)	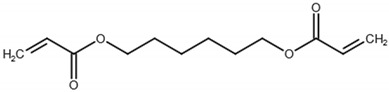
Rose Bengal sodium salt (RB)	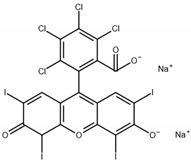
Eosin Y (EY)	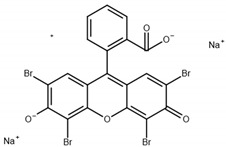
Methylene Blue (MB)	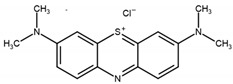

## Data Availability

Not applicable.

## Supplementary Materials

The following are available online at https://www.mdpi.com/article/10.3390/gels8030156/s1, Figure S1: UV-visible absorption spectra of the three dyes at different concentrations (solid line: C = 0.064 mg/mL, dashed line: C = 0.032 mg/mL); Figure S2: UV-visible absorption spectra of RB solutions with a contact time of 24 h; Figure S3: UV absorption spectra of MB solutions with a contact time of 24 h; Figure S4: UV-visible absorption spectra of EY solutions with a contact time of 24 h; Figure S5: Representation of crosslinked poly(acrylamide-*co*-acrylic acid)/HDDA; Table S1: Definition of Abbreviation; Table S2: Grid box center dimension for all systems.
