# Supplementary material for: Cleaning of Wastewater Using Crosslinked Poly(Acrylamide-co-Acrylic Acid) Hydrogels: Analysis of Rotatable Bonds, Binding Energy and Hydrogen Bonding
Source: Gels. 2022 Mar 3;8(3):156. doi: 10.3390/gels8030156 (PMC8952127; doi:10.3390/gels8030156)
Supplement: Supplementary file 1 [file gels-08-00156-s001.zip › gels-1605888-supplementary.pdf]

Supplementary Material

Cleaning of wastewater using crosslinked poly(acrylamide-co-acrylic acid) hydrogels: Analysis of rotatable bonds, binding energy and hydrogen bonding

Salah Hamri, Tewfik Bouchaour, Djahida Lerari, Zohra Bouberka, Philippe Supiot and Ulrich Maschke

Hydrogels: experimental procedure

First step:
The stock solution of AA was prepared, by dissolving 20g of AA monomer (powder) in 150mL of distilled water. The concentration of AA was thus 0.13 g/mL. The solution was stirred for 24 hours.
Second step:
To elaborate poly(AM) with different concentrations of HDDA, three reagent solutions were prepared as follows:
Solution 1: 98.5 wt% of stock solution of AA, 0.5 wt% Darocur 1173, 1 wt% HDDA.
Solution 2: 95.5 wt% of stock solution of AA, 0.5 wt% Darocur 1173, 4 wt% HDDA.
Solution 3: 92.5 wt% of stock solution of AA, 0.5 wt% Darocur 1173, 7 wt% HDDA.
Third step:
The stock solution of AM/AA was prepared with:
50 wt% stock solution of AM and 50 wt% AA
Fourth step:
To elaborate poly(AM-co-AA) with different concentrations of HDDA, three reagent solutions were prepared as follows:
Solution 4: 98.5 wt% of stock solution of AM/AA, 0.5 wt% Darocur 1173, 1 wt% HDDA.
Solution 5: 95.5 wt% of stock solution of AM/AA, 0.5 wt% Darocur 1173, 4 wt% HDDA.
Solution 6: 92.5 wt% of stock solution of AM/AA, 0.5 wt% Darocur 1173, 7 wt% HDDA.

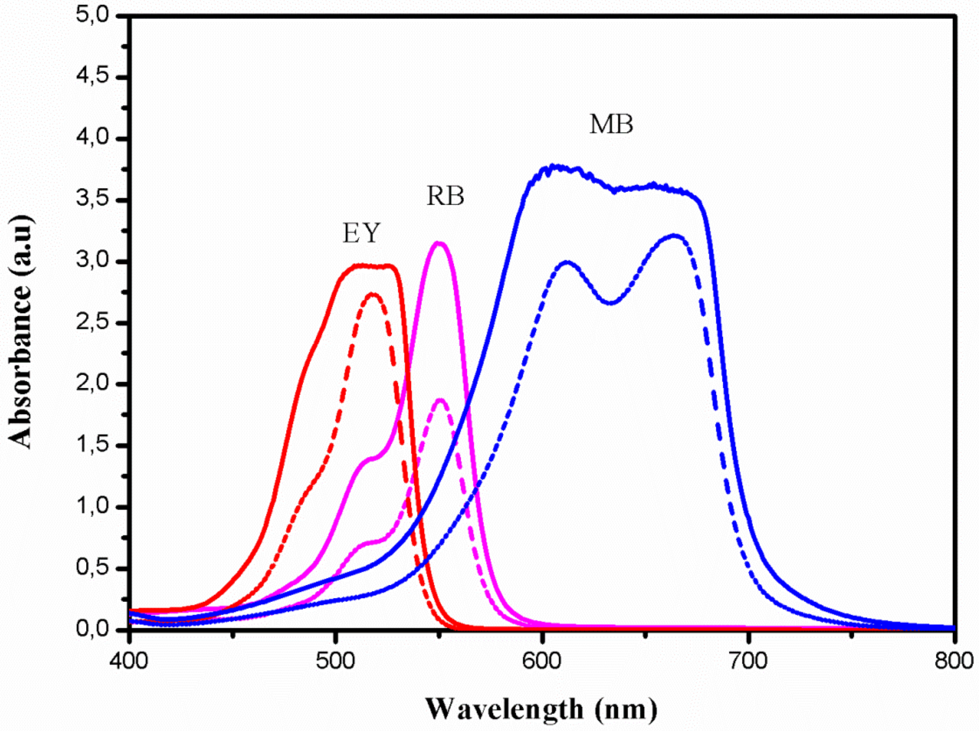

Figure S1. UV-visible absorption spectra of the three dyes at different concentrations (solid line: C = 0.064mg/mL, dashed line: C = 0.032mg/mL).

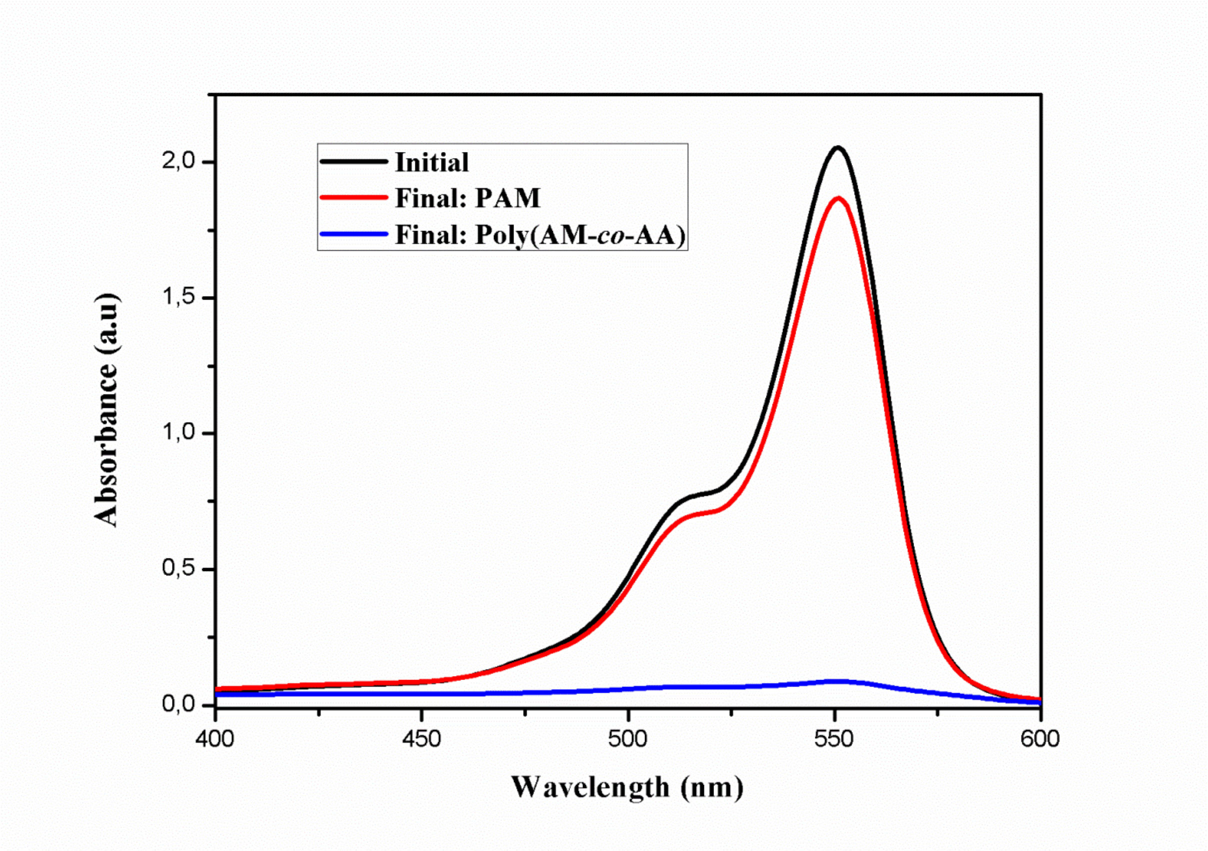

Figure S2. UV-visible absorption spectra of RB solutions with a contact time of 24 h.

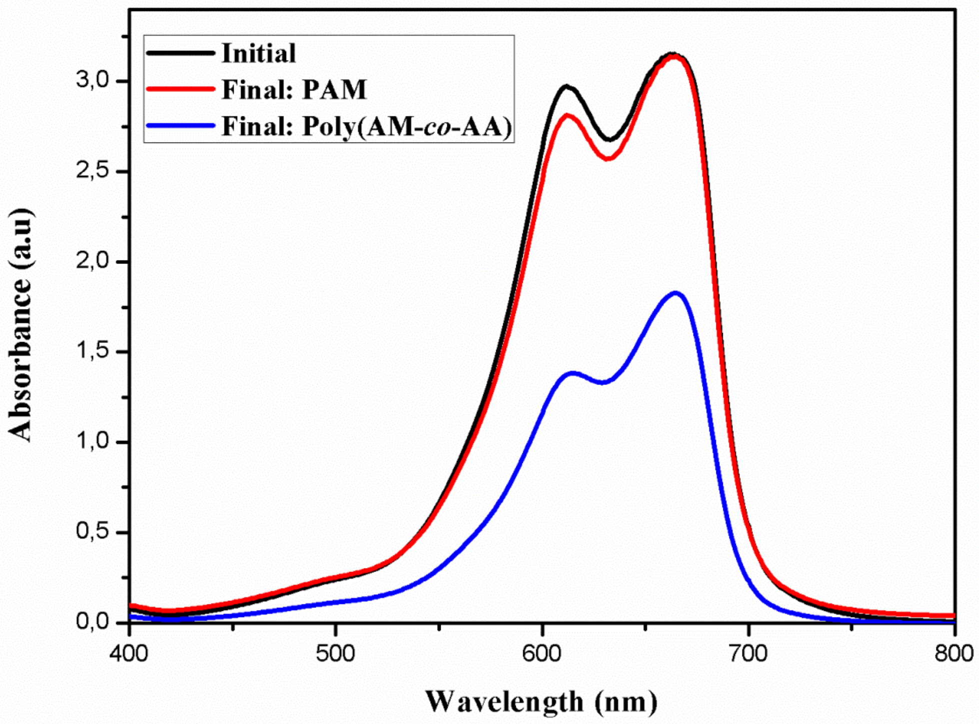

Figure S3. UV absorption spectra of MB solutions with a contact time of 24 h.

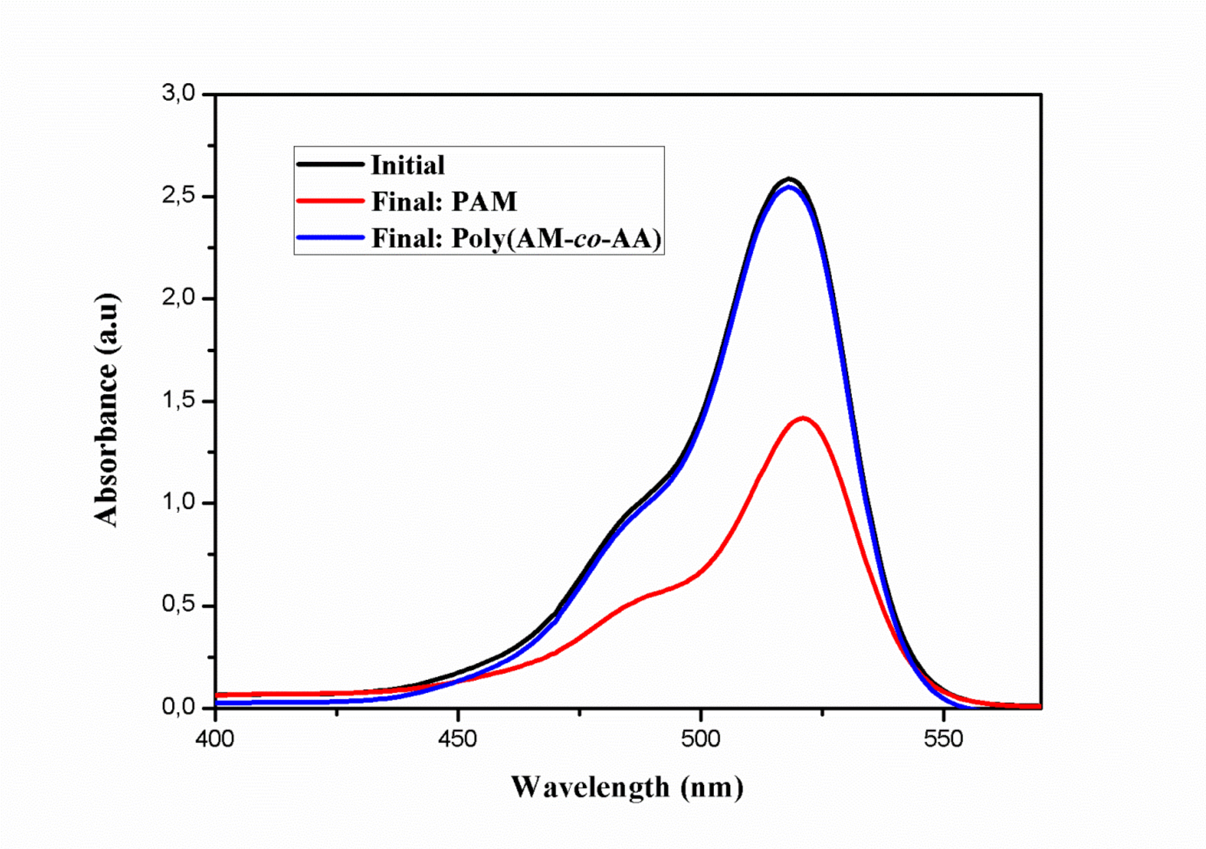

Figure S4. UV-visible absorption spectra of EY solutions with a contact time of 24 h.

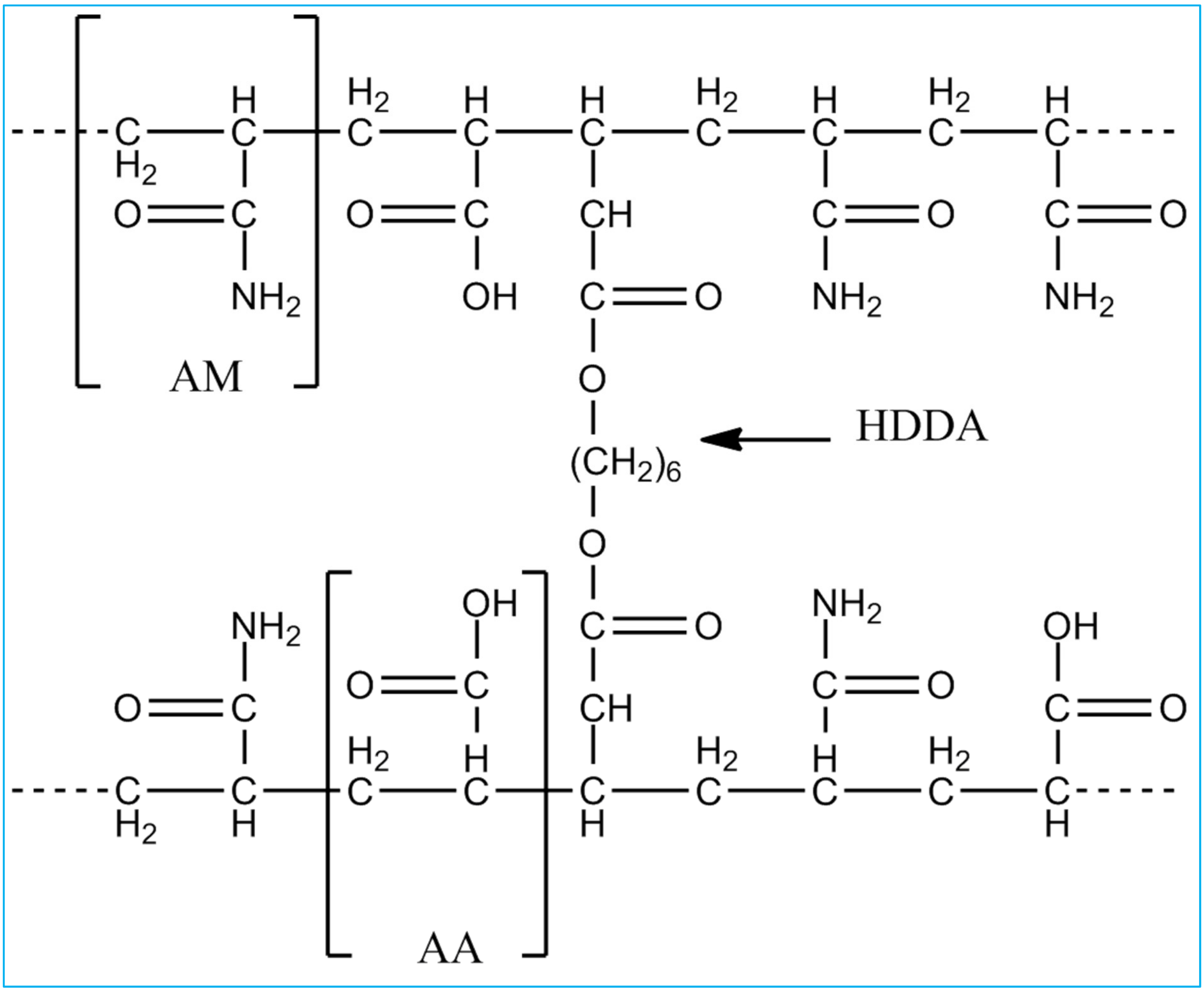

Figure S5. : Representation of crosslinked poly(acrylamide-*co*-acrylic acid)/HDDA.

Table S1. Definition of Abbreviation.

Abbreviation	Definition
AM	Acrylamide
PAM	Polyacrylamide
AA	Acrylic acid
HDDA	1,6-HexaneDiolDiAcrylate
HEMA	Hydroxyethyl methacrylate
NMBAM	N,N′-Methylene bisacrylamide
RB	Rose Bengal
MB	Methylene blue
EY	Eosin Y
UV	Ultraviolet

Table S2. Grid box center dimension for all systems.

Polymer/dye	Grid box center dimensions (Å)		
	x	y	z
Poly(AM)/RB	-18.473	-9.776	-35.491
Poly(AM)/EY	-18.473	-9.776	-35.491
Poly(AM)/MB	-18.473	-9.776	-35.491
Poly(AM-*co*-AA)/RB	-21.178	-10.397	-40.171
Poly(AM-*co*-AA)/EY	0.111	-0.639	-52.250
Poly(AM-*co*-AA)/MB	-20.778	-2.306	-28.750
